# Awake brain MRSI reveals anesthetic sensitivity and regional aging effects on [^13^C]bicarbonate metabolism in mice

**DOI:** 10.3389/fnimg.2025.1506126

**Published:** 2025-02-12

**Authors:** Maiko Ono, Rena Kono, Kosei Hirata, Keita Saito, Motonao Nakao, Yoichi Takakusagi, Rikita Araki, Akira Sumiyoshi, Yuhei Takado

**Affiliations:** ^1^Institute for Quantum Life Science, National Institutes for Quantum Science and Technology, Chiba, Japan; ^2^Department of Neuroscience, Genetics, and Genomic Sciences, Icahn School of Medicine at Mount Sinai, New York, NY, United States; ^3^Department of Neurology and Neurological Science, Tokyo Medical and Dental University, Tokyo, Japan; ^4^Biospin Division, Application Department, Bruker Japan K.K., Yokohama, Japan; ^5^Institute for Quantum Medical Science, National Institutes for Quantum Science and Technology, Chiba, Japan

**Keywords:** hyperpolarized [1-13C]pyruvate, carbon-13 MRS, chemical shift imaging, mouse brain, awake condition, aging, bicarbonate

## Abstract

Abnormalities and alterations in the glycolytic pathway in the pathology of neurodegenerative diseases and brain aging have received much attention, as clinical applications of proton-based magnetic resonance spectroscopy (MRS) have recently illuminated the elevation of lactate concentrations in the brains of patients with neurodegenerative diseases, including Alzheimer’s disease. Hyperpolarized [1-^13^C]pyruvate MRS has shown promise for neurological applications because it enables the real-time *in vivo* detection of glycolysis and oxidative phosphorylation flux. In studies of the mouse brain using hyperpolarized [1-^13^C]pyruvate, there are few reports that the signal of [^13^C]bicarbonate, a product of oxidative phosphorylation metabolized from [1-^13^C]pyruvate, was detected using MR spectroscopic imaging (MRSI) that allows spatial mapping of metabolism, although there have been reports of [^13^C]bicarbonate signals being detected by pulse-acquire sequences in the entire brain. In the present study, we compared hyperpolarized [1-^13^C]pyruvate metabolism between the brains of awake and isoflurane-anesthetized mice using a custom-made awake mouse restraint device with MRSI. Although the signal for [1-^13^C]lactate, a product of glycolysis metabolized from [1-^13^C]pyruvate, was detectable in multiple brain regions that include the orbitofrontal cortex and hippocampus in both awake and anesthetized mice, the signal for [^13^C]bicarbonate metabolized from [1-^13^C]pyruvate was only detectable in the brains of awake mice. Moreover, a comparison of hyperpolarized [1-^13^C]pyruvate metabolism in young and aged mouse brains using awake MRSI detected age-related decreases in oxidative phosphorylation flux in brain regions that include the hippocampus with variations in the extent of these changes across different brain regions. These results demonstrate that hyperpolarized [1-^13^C]pyruvate MRSI under awake conditions is useful for the spatial detection of abnormalities and alterations in glycolysis and oxidative phosphorylation flux in the brains of mice. Thus, the use of hyperpolarized [1-^13^C]pyruvate MRSI has potential in pathological and mechanistic studies of brain diseases and brain aging.

## 1 Introduction

Magnetic resonance spectroscopy (MRS) can distinguish between several individual metabolites based on their chemical shifts, allowing for the non-invasive assessment of the metabolic state of tissues. Abnormalities and alterations in the glycolytic pathway in the pathology of neurodegenerative diseases and brain aging have received much attention, as clinical applications of proton-based MRS have recently illuminated the elevation of lactate concentrations in the brains of patients with Alzheimer’s disease and progressive supranuclear palsy (Hirata et al., 2024a, 2024b). ^13^C MRS provides detailed information about metabolic flux, owing to its ability to provide information on the conversion of injected ^13^C-labeled substrates to metabolites (Shulman and Rothman, 2001). However, the low sensitivity and relatively low concentrations of metabolites, which make spatial mapping of metabolism almost impossible, have been a weakness of ^13^C MRS. Recently, hyperpolarization methods, such as dissolution dynamic nuclear polarization (dDNP), have improved the transient sensitivity of ^13^C-labeled substrates and metabolites by more than 10,000-fold, making it possible to detect and image their metabolic flux in real time using ^13^C MRS (Ardenkjaer-Larsen et al., 2003; Eichhorn et al., 2013).

Pyruvate plays a central role in the glycolytic pathway as a crossing point between anaerobic and aerobic metabolism at the end of the pathway. The neurological applications of hyperpolarized (HP) [1-^13^C]pyruvate MRS have shown great promise, and preclinical development and clinical applications are currently underway (Le Page et al., 2020; Li et al., 2021; Chaumeil et al., 2024). Human studies using HP [1-^13^C]pyruvate suggest that brain pyruvate metabolism through the formation of both lactate and bicarbonate, products of glycolysis and oxidative phosphorylation, respectively, is systematically observed (Park et al., 2018; Grist et al., 2019, 2020; Hackett et al., 2020; Lee et al., 2020). In a mouse brain study using HP [1-^13^C]pyruvate, there were few instances whereby the signal of [^13^C]bicarbonate metabolized from [1-^13^C]pyruvate was detected using MR spectroscopic imaging (MRSI) that allowed spatial mapping of metabolism, although there have been reports of signals of [^13^C]bicarbonate metabolized from [1-^13^C]pyruvate being detected using pulse-acquire sequences in the entire brain (Eichhorn et al., 2013; Choi et al., 2018). One potential explanation for the differences between species in the detection of HP [1-^13^C]pyruvate metabolite signals in the brain is anesthesia. In clinical HP ^13^C MRS studies, patients are usually awake, whereas in preclinical studies, animals are anesthetized for immobilization. However, anesthesia can have profound effects on energy metabolism. The side effects of isoflurane anesthesia include depressed heart and breathing rates, dose-dependent vasodilation leading to elevated cerebral blood flow (CBF), and depression of brain metabolism (Lukasik and Gillies, 2003; Josan et al., 2013; Hyppönen et al., 2022). A previous study comparing the metabolic states of HP [1-^13^C]pyruvate in the rat brain under awake with anesthetized conditions, using slice-selective pulse imaging, showed that pyruvate-bicarbonate and pyruvate-lactate labeling rates were lower in isoflurane-anesthetized animals than in awake animals (Hyppönen et al., 2022).

The aim of this study was to investigate the effects of anesthetic agents and aging on [^13^C]bicarbonate metabolism in the mouse brain using hyperpolarized [1-^13^C]pyruvate MRSI under awake conditions. We sought to determine the feasibility of detecting [^13^C]bicarbonate signals in specific brain regions and to explore the regional and age-dependent variations in oxidative phosphorylation flux.

## 2 Materials and methods

### 2.1 Animals

All animal procedures were performed according to the National Research Council Guide for the Care and Use of Laboratory Animals. The protocols for animal experiments were approved by the Animal Ethics Committee of the National Institutes for Quantum Science and Technology (approval number: 22-1012-4). Ten adult male C57BL/6 N mice (Japan SLC, Inc., Shizuoka, Japan) and 18 adult C57BL/6J mice (male, The Jackson Laboratory Japan, Inc., Kanagawa, Japan) were used for the MR experiments in this study (Supplementary tables). All mice were maintained in a 12-h light/dark cycle with *ad libitum* access to a standard diet and water.

**Figure 1:**
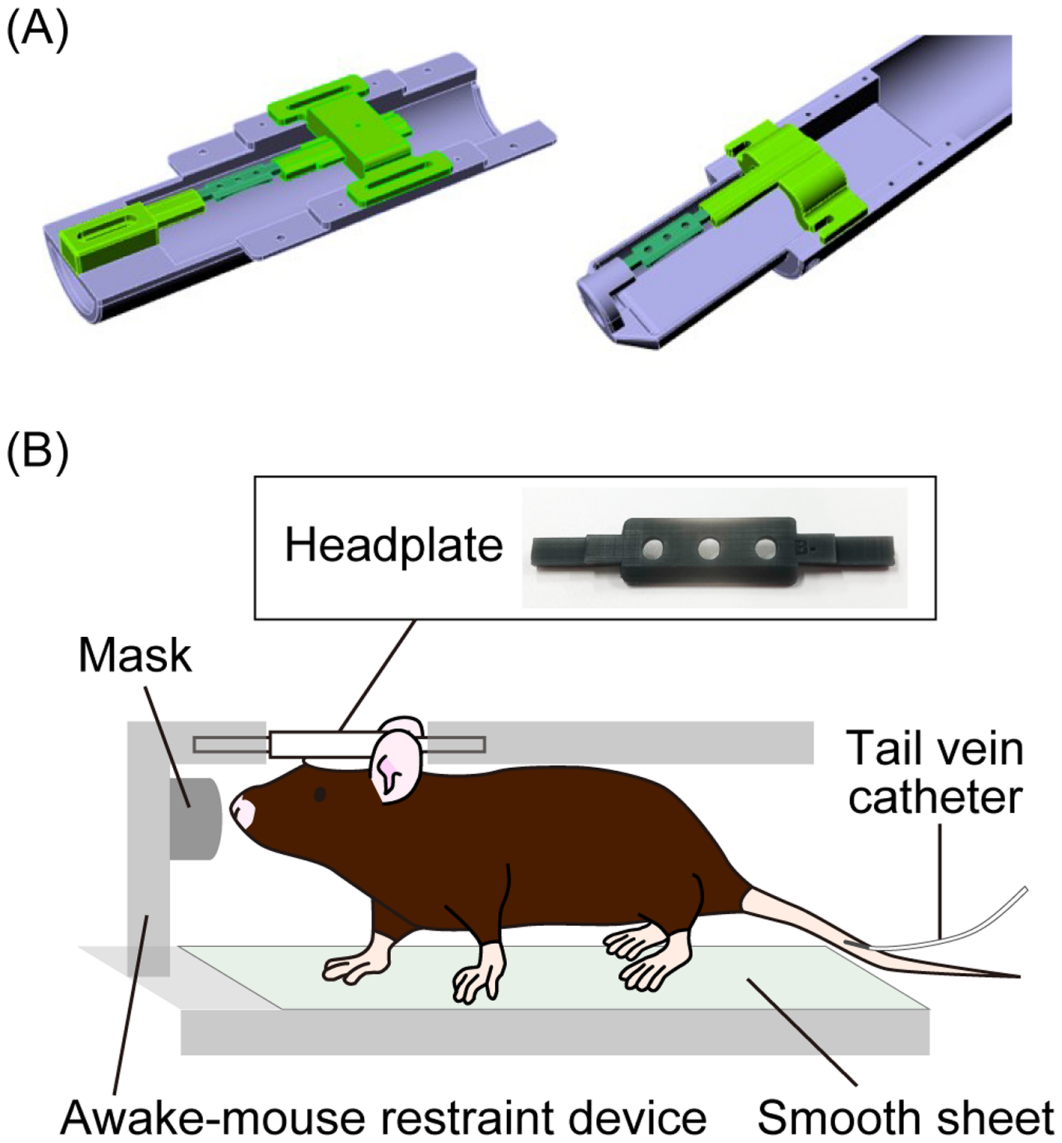
**(A)** Graphic images of a custom-made awake mouse restraint device for a 3 T (left) and 7 T (right) preclinical magnetic resonance (MR) scanner. **(B)** A headplate placed on the cranium of a mouse and a schematic diagram of a mouse placed in the awake mouse restraint device for *in vivo* hyperpolarized ^13^C MR spectroscopy (MRS).

To compare the brain metabolism of HP [1-^13^C]pyruvate in awake and isoflurane-anesthetized mice, we developed a measurement system by making an awake mouse restraint device and a head plate to place the mice on the device (Figure 1; Supplementary Figure S1). For the surgical procedure, the animals were anesthetized with a mixture of air, oxygen, and isoflurane (2% w/v for induction and 1% w/v for surgery) via a facemask, and a custom-made headplate (Figure 1) was attached to the cranium using a dental bond (Gluma Bond Universal, Kulzer GmbH, Hanau, Germany) and resin (LuxaFlow Star, DMG Chemisch-Pharmazeutische Fabrik GmbH, Hamburg, Germany). The animals were anesthetized with 1.5% (w/v) isoflurane, and a tail vein catheter was inserted for the injection of HP [1-^13^C]pyruvate (Supplementary Figure S2). The mice were anesthetized with 1–2% (w/v) isoflurane for less than 20 min during cannulation and positioning in the magnetic resonance imaging (MRI)/MRS-compatible awake mouse restraint device via the headplate (Figure 1; Supplementary Figure S2), and the device was subsequently inserted inside the scanner. During MRI/MRS under anesthesia, 1.5 ± 0.3% (w/v) isoflurane was supplied to mice through a mask installed on the awake mouse restraint device, and mouse physiology was monitored. The body temperature was stabilized between 37 and 38°C, while the respiration rate was maintained at 80–100 min^−1^ by adjusting the isoflurane dose. For awake mouse experiments, the isoflurane supply to the mice was turned off for at least 5 min before imaging. Imaging was performed after confirming that the mice were awake using a respiration monitor.

### 2.2 Pyruvate hyperpolarization

[1-^13^C]Pyruvic acid (18 μL; Cat# 677175; Sigma-Aldrich, St Louis, MO, United States), containing 15 mmol/L of OX63, was hyperpolarized at 1.3 K, 6.7 T, and 187.77 GHz for 1 h in a SpinAligner (Polarize Aps, Frederiksberg, Denmark) according to the manufacturer’s instructions. The hyperpolarized sample was rapidly dissolved in 3.2 mL of a superheated alkaline buffer consisting of 40 mmol/L tris(hydroxymethyl)aminomethane (Cat# 819620, MP Biomedicals, Irvine, CA, United States), 50 mmol/L of sodium chloride (Cat# 191-01665, FUJIFILM Wako, Osaka, Japan), 0.27 mmol/L of EDTA (Cat# 195173, MP Biomedicals, Irvine, CA, United States), and 80 mmol/L of sodium hydroxide (Cat# 31511-05, Nacalai Tesque, Kyoto, Japan). The HP [1-^13^C]pyruvate solution (80.7 mmol/L) was confirmed to be near pH 7, and was intravenously injected into mice through a tail vein catheter (10 μL/g body weight).

### 2.3 *In vivo* hyperpolarized ^13^C MRS and imaging

All hyperpolarized MR studies were performed using a BioSpec 3 T preclinical MR scanner (Bruker Biospin) with a ^1^H-^13^C dual-tuned surface transmit/receive coil. The ^13^C coil was loop-shaped (18 × 22 mm) and bent along its 18 mm axis to conform to a cylindrical surface with a diameter of 24 mm. The ^1^H coil was butterfly-shaped (32 × 24 mm) and bent along its 32 mm axis to fit the same cylindrical surface (diameter: 24 mm). After the mouse had been positioned inside the magnet, a series of axial, sagittal, and coronal two-dimensional images were acquired using a RARE sequence (TR = 2,400 ms, TE = 12 ms, RARE factor = 10, number of averages = 4, number of repetitions = 1, field of view = 24 × 24 mm, matrix = 192 × 192, flip angle = 90°). A volume of interest (VOI) for pulse-acquire sequences or a slab for MRSI was positioned across the coronal plane to cover the whole brain (Supplementary Figure S3). The targeted regions were shimmed to reduce the localized proton linewidth to 20 Hz, using the B0 map-based shim and local shim sequences provided by Bruker. For pulse-acquire sequences, the acquisition was started at the end of the dissolution, and non-selective RF pulse ^13^C acquisitions were sequentially recorded every 3 s using 10° radiofrequency pulses. Localization was achieved by placing a surface coil on top of the mouse’s head. For the analysis of the pulse-acquire sequences, Fourier transformation was applied to the free induction decay (FID) to generate spectra, which were displayed in absorption mode using Mnova (Mestrelab Research, Galicia, Spain). An apodization function with a 2 Hz exponential line broadening was applied, followed by automatic phase correction. A total of 30 peaks, beginning with the onset of the first pyruvate peak, were integrated, and their peak heights were quantified. To map metabolites, starting 4 s after the injection of HP [1-^13^C]pyruvate, HP ^13^C FID chemical shift images were obtained with a flip angle of 10°, a TR of 85 ms, a TE of 1.245 ms, and five repetitions from an 8 mm coronal slice of the brain. The field of view was 24 × 24 mm^2^ with a matrix size of 12 × 12, resulting in a total scan time of 61 s. An HP ^13^C metabolite map was produced by measuring the peak value of each metabolite and overlaying it on a proton T2-weighted image. The spectrum for quantification was obtained by summing all five repetitions. The MRSI data were analyzed in both magnitude mode and absorption modes (Supplementary Figures S4, S5) using custom-built programs written in MATLAB (MathWorks, Natick, MA, United States). After evaluating the advantages of each analysis method, the results were displayed in magnitude mode. Specifically, the FID data were subjected to Fourier transformation, and spectra from the regions of interest were extracted. When spectra were obtained from multiple regions, they were summed. Finally, the relative ratios of individual metabolites were quantified based on their peak heights.

### 2.4 Cerebral perfusion measurements

MRI data were acquired using a horizontal 7.0 T Bruker BioSpec 70/40 MRI system with an 86 mm volume transmit and a 4-channel phased array receive-only cryoprobe (Bruker Biospin, Ettlingen, Germany). The software and console of the MRI scanner used were ParaVision 360 and AVANCE NEO, respectively. Following standard adjustment routines, pilot scans (tripilot sequence) were used to accurately position the animal head inside the magnet. CBF images were obtained using a 2D flow-sensitive alternating inversion recovery RARE pulse sequence with the following parameters: inversion recovery time = 20, 200, 300, 500, 800, 1,400, 1,700, and 2,000 ms, repetition time = 12,000 ms, effective echo time = 44 ms, RARE factor = 16, field of view = 24 × 16 mm^2^, matrix size = 96 × 64, in-plane resolution = 0.25 × 0.25 mm^2^, number of slices = 1, slice thickness = 1 mm, and number of averages = 1. CBF data analysis (Sumiyoshi et al., 2022) was performed using software custom-written in MATLAB (MathWorks, Natick, MA, United States), which consisted of calculations of the selective T1 map (T1_sel_), global T1 map (T1_nonsel_), and perfusion map (CBF). The calculations for T1_sel_ and T1_nonsel_ were performed using non-linear least-squares fit to the data for each voxel in the images with different inversion recovery times (eight images each). CBF was calculated from the measurements of T1_sel_ and T1_nonsel_, which were obtained using the equation: CBF (mL/100 g/min) = *λ* * T1_nonsel_/T1_blood_ * (1,000/T1_sel_ – 1,000/T1_nonsel_), where λ is the blood–brain partition coefficient, i.e., the ratio between the water concentration per gram of brain tissue and per milliliter of blood. λ was set to 4,980. T1_blood_ was set to 2.3, which was derived from the measurements of rat blood at 7.0 T. CBF images were evaluated for the region of interest (region placement is displayed in Supplementary Figure S6) using PMOD image analysis software (PMOD Technologies Ltd.).

### 2.5 Statistics

The data were analyzed using GraphPad Prism version 6.0 (GraphPad, San Diego, CA, United States). Unpaired t-tests were performed to compare two groups, assuming equal group variances (*p* < 0.05). The results are reported as the mean ± standard deviation.

## 3 Results

An awake mouse restraint device was designed to supply isoflurane through the mask. [1-^13^C]Lactate signals were consistently observed following [1-^13^C]pyruvate injection in awake and isoflurane anesthesia protocols using an awake mouse restraint device. Therefore, the constructed measurement system was deemed suitable for the dDNP experiments.

**Figure 2:**
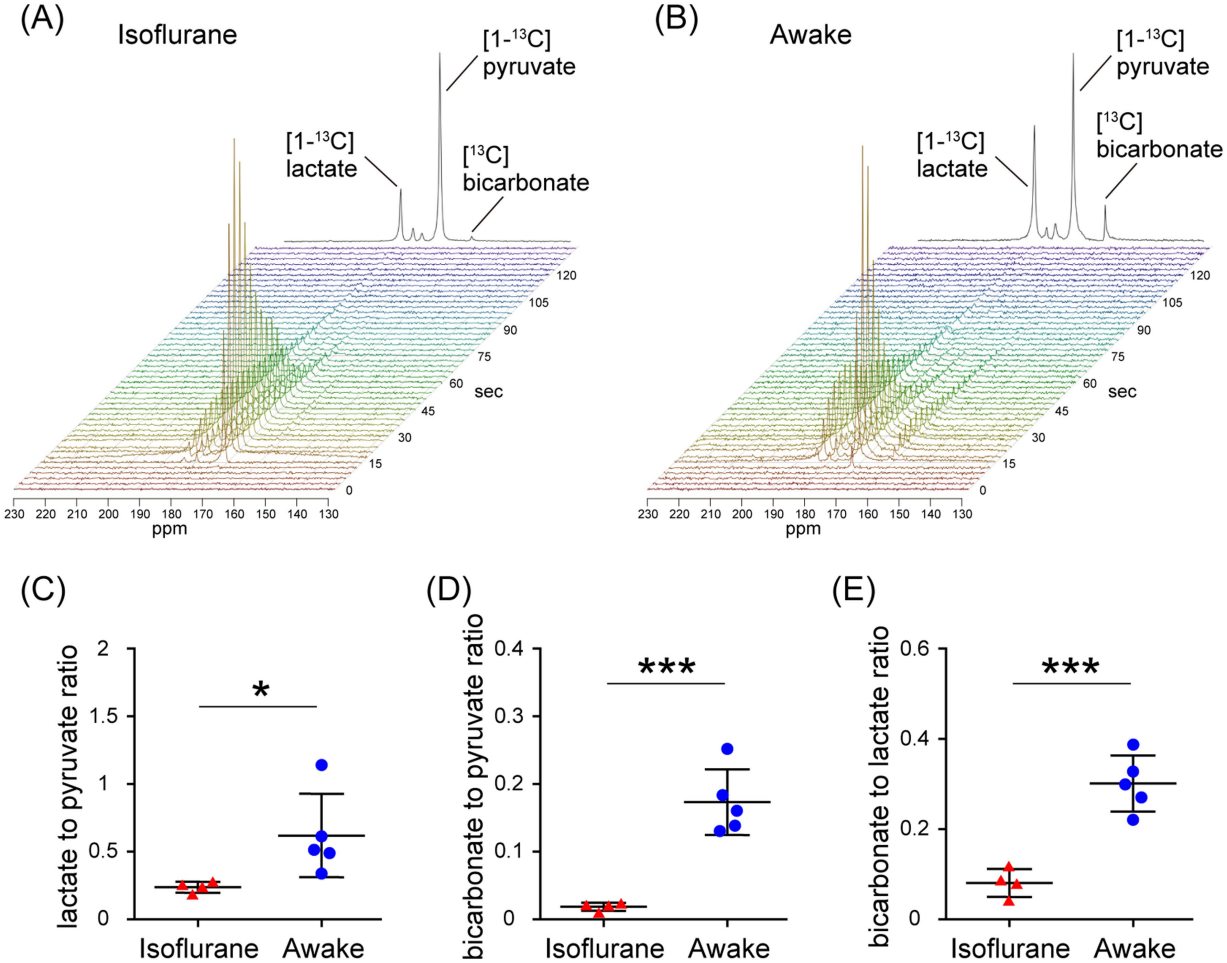
*In vivo* real-time pyruvate metabolism evaluated using pulse-acquire sequences in isoflurane-anesthetized and awake C57BL/6 N mouse brains. *In vivo* ^13^C MR data recording was initiated prior to the injection of a [1-^13^C]pyruvate solution (80.7 mmol/L, 10 μL/g body weight). **(A,B)** Series of ^13^C spectra recorded every 3 s after acquisition start (lower, display from 0 to 135 s) and the sum of 30 peaks (upper), beginning with the onset of the first pyruvate peak, in the brain of 2-month-old isoflurane-anesthetized **(A)** and awake **(B)** C57BL/6 N mice. **(C–E)** Quantification of the [1-^13^C]lactate to [1-^13^C]pyruvate ratio **(C)**, the [^13^C]bicarbonate to [1-^13^C]pyruvate ratio **(D)**, and the [^13^C]bicarbonate to [1-^13^C]lactate ratio **(E)** in the brains of 2-month-old isoflurane-anesthetized (*n* = 4) and awake (*n* = 5) C57BL/6 N mice. *p < 0.05, ***p < 0.0005.

**Figure 3:**
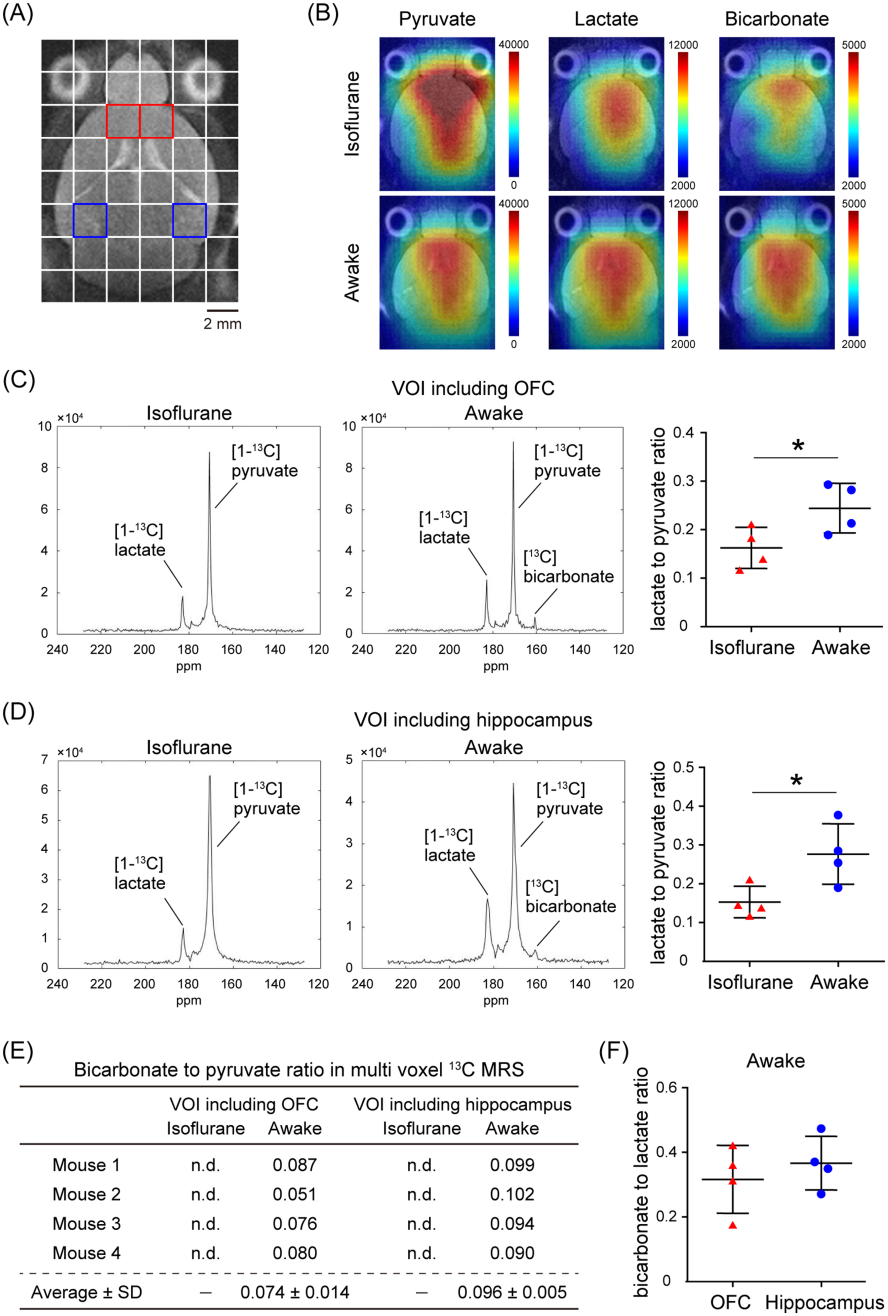
*In vivo* real-time pyruvate metabolism evaluated using MRSI in isoflurane-anesthetized and awake C57BL/6 N mouse brains. *In vivo* ^13^C MR data were recorded following the injection of a [1-^13^C]pyruvate solution (80.7 mmol/L, 10 μL/g body weight). **(A)** A horizontal T2-weighted MR image of a C57BL/6 N mouse brain, with red and blue squares indicating representative volumes of interest (VOIs) that include the orbitofrontal cortex (OFC) and hippocampus, respectively. **(B)** Representative metabolic maps for [1-^13^C]pyruvate, [1-^13^C]lactate, and [^13^C]bicarbonate in isoflurane-anesthetized (upper) and awake (lower) C57BL/6 N mouse brains, superimposed on a horizontal T2-weighted MR image. **(C)** Summed ^13^C spectra from 0 to 60 s after acquisition start in the VOIs that include the OFC in 2-month-old isoflurane-anesthetized (left) and awake (middle) C57BL/6 N mice, with quantification of the [1-^13^C]lactate to [1-^13^C]pyruvate ratio in the VOIs that include the OFC of 2-month-old isoflurane-anesthetized (*n* = 4) and awake (*n* = 4) C57BL/6 N mice. **(D)** Summed ^13^C spectra from 0 to 60 s after acquisition start in the VOIs that include the hippocampus in 2-month-old isoflurane-anesthetized (left) and awake (middle) C57BL/6 N mouse, with quantification of the [1-^13^C]lactate to [1-^13^C]pyruvate ratio in the VOIs that include the hippocampus of 2-month-old isoflurane-anesthetized (*n* = 4) and awake (*n* = 4) C57BL/6 N mice. **(E)** Quantification of the [^13^C]bicarbonate to [1-^13^C]pyruvate ratio in the VOIs that include the OFC and hippocampus in 2-month-old isoflurane-anesthetized (*n* = 4) and awake (*n* = 4) C57BL/6 N mice. **(F)** Quantification of the [^13^C]bicarbonate to [1-^13^C]lactate ratio in the VOIs that include the OFC and hippocampus of 2-month-old awake (*n* = 4) C57BL/6 N mice. *p < 0.05.

**Figure 4:**
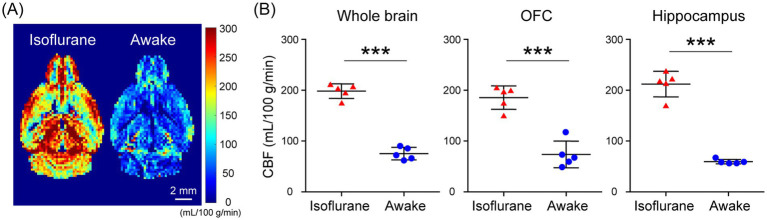
Cerebral blood flow (CBF) evaluated using flow-sensitive alternating inversion recovery (FAIR) arterial spin labeling techniques in isoflurane-anesthetized and awake C57BL/6 N mouse brains. **(A)** Representative perfusion maps of 4-month-old isoflurane-anesthetized and awake C57BL/6 N mice. **(B)** Quantification of CBF in the whole brain (left), OFC (middle), and hippocampus (right) of 4-month-old isoflurane-anesthetized (*n* = 5) and awake (*n* = 5) C57BL/6 N mice. ***p < 0.0005.

In the assessment of pulse-acquire sequences, the signals of [1-^13^C]lactate and [^13^C]bicarbonate metabolized from [1-^13^C]pyruvate were detectable in both 1.5 ± 0.3% (w/v) isoflurane-anesthetized and awake mouse brains (Figures 2A,B). The ratios of [1-^13^C]lactate to [1-^13^C]pyruvate, [^13^C]bicarbonate to [1-^13^C]pyruvate, and [^13^C]bicarbonate to [1-^13^C]lactate were significantly higher in awake mice compared to isoflurane-anesthetized mice (Figures 2C–E; [1-^13^C]lactate to [1-^13^C]pyruvate ratio: *p* = 0.0451; [^13^C]bicarbonate to [1-^13^C]pyruvate ratio: *p* = 0.0004; [^13^C]bicarbonate to [1-^13^C]lactate ratio: *p* = 0.0004). Regarding MRSI, the signal of [1-^13^C]lactate metabolized from [1-^13^C]pyruvate was detectable in multiple brain regions that include the orbitofrontal cortex (OFC) and hippocampus in both 1.5 ± 0.3% (w/v) isoflurane-anesthetized and awake mice (Figures 3A–D). The [1-^13^C]lactate to [1-^13^C]pyruvate ratio was significantly higher in awake mice than in isoflurane-anesthetized mice in brain regions that include the OFC (*p* = 0.0483) and hippocampus (*p* = 0.0304) in assessments using MRSI, consistent with the findings from pulse-acquire sequences assessment. On the other hand, the signal of [^13^C]bicarbonate metabolized from [1-^13^C]pyruvate was below the detection limit in brain regions that include the OFC and hippocampus of 1.5 ± 0.3% (w/v) isoflurane-anesthetized mice, while it was detectable in awake mouse brains (Figures 3B–E). No significant differences were observed in the [^13^C]bicarbonate to [1-^13^C]lactate ratio between brain regions that include the OFC and hippocampus in assessments using MRSI (Figure 3F: *p* = 0.4816). At this time, CBF was significantly higher in the whole brain (*p* < 0.0001), the OFC (*p* < 0.0001), and the hippocampus (*p* < 0.0001) of 1.5 ± 0.3% (w/v) isoflurane-anesthetized mice compared to awake mice (Figures 4A,B).

**Figure 5:**
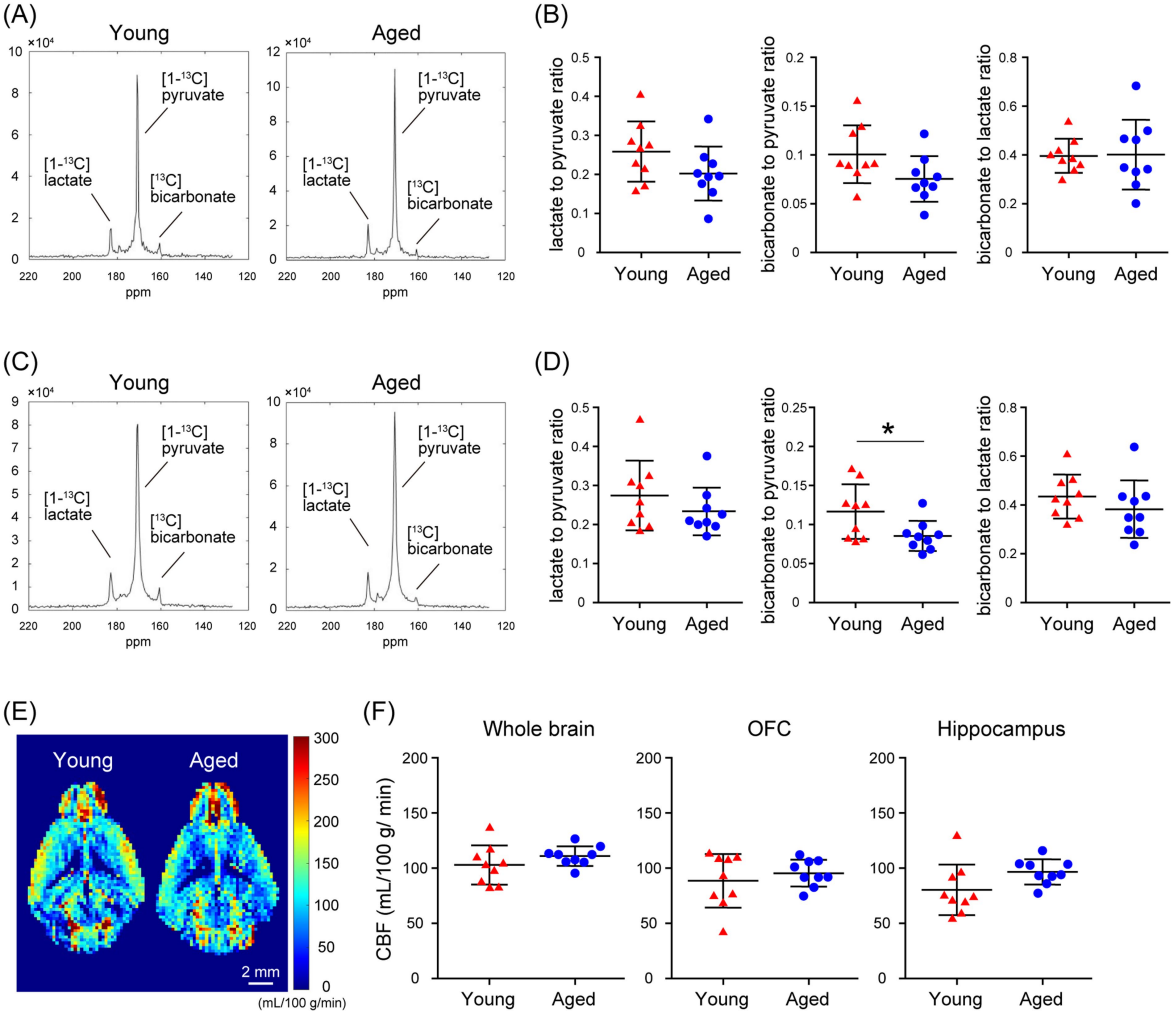
Comparison of *in vivo* real-time pyruvate metabolism in the brains of young and aged C57BL/6J mice as evaluated using MRSI under awake conditions. *In vivo* ^13^C MR data were recorded following the injection of a [1-^13^C]pyruvate solution (80.7 mmol/L, 10 μL/g body weight). **(A)** Summed ^13^C spectra from 0 to 60 s after acquisition start in the VOIs that include the OFC of 2.5-month-old (young, left) and 19-month-old (aged, right) C57BL/6J mice. **(B)** Quantification of the [1-^13^C]lactate to [1-^13^C]pyruvate ratio (left), the [^13^C]bicarbonate to [1-^13^C]pyruvate ratio (middle), and the [^13^C]bicarbonate to [1-^13^C]lactate ratio (right) in the VOIs that include the OFC of 2.5-month-old (young, *n* = 9) and 19-month-old (aged, *n* = 9) C57BL/6J mice. **(C)** Summed ^13^C spectra from 0 to 60 s after acquisition start in the VOIs that include the hippocampus of 2.5-month-old (young, left) and 19-month-old (aged, right) C57BL/6J mice. **(D)** Quantification of the [1-^13^C]lactate to [1-^13^C]pyruvate ratio (left), the [^13^C]bicarbonate to [1-^13^C]pyruvate ratio (middle), and the [^13^C]bicarbonate to [1-^13^C]lactate ratio (right) in the VOIs that include the hippocampus of 2.5-month-old (young, *n* = 9) and 19-month-old (aged, *n* = 9) C57BL/6J mice. **(E)** Representative perfusion maps from 2.5-month-old (young) and 19-month-old (aged) awake C57BL/6J mouse brains. **(F)** Quantification of CBF in the whole brain (left), OFC (middle), and hippocampus (right) of 2.5-month-old (young, *n* = 9) and 19-month-old (aged, *n* = 9) awake C57BL/6J mouse. *p < 0.05.

Using MRSI in awake mice, which allows spatial mapping of the metabolism of HP [1-^13^C]pyruvate to lactate and bicarbonate, we evaluated the alterations in glycolysis and oxidative phosphorylation flux with aging in the mouse brain. No significant differences were detected in the [1-^13^C]lactate to [1-^13^C]pyruvate or the [^13^C]bicarbonate to [1-^13^C]lactate ratio in brain regions that include the OFC ([1-^13^C]lactate to [1-^13^C]pyruvate ratio: *p* = 0.1229; [^13^C]bicarbonate to [1-^13^C]lactate ratio: *p* = 0.9246) and hippocampus ([1-^13^C]lactate to [1-^13^C]pyruvate ratio: *p* = 0.2739; [^13^C]bicarbonate to [1-^13^C]lactate ratio: *p* = 0.3097), between young (2.5-months-old) and aged mice (19-months-old; Figures 5A–D). There was no significant difference in the [^13^C]bicarbonate to [1-^13^C]pyruvate ratio in brain regions that include the OFC between young and aged mice (*p* = 0.0618). However, the [^13^C]bicarbonate to [1-^13^C]pyruvate ratio in brain regions that include the hippocampus was significantly lower in aged mice than in young mice (*p* = 0.0323; Figures 5A–D). At this time, CBF in the whole brain, the OFC, and the hippocampus of awake C57BL/6J mice did not differ significantly between young and aged mice (Figures 5E,F; whole brain: *p* = 0.2369, OFC: *p* = 0.4568, hippocampus: *p* = 0.0752). These results demonstrated that MRSI evaluation of HP [1-^13^C]pyruvate metabolism in awake mice is useful for the spatial detection of abnormalities and alterations in glycolysis in the brains of mice.

## 4 Discussion

In the present study, HP [1-^13^C]pyruvate metabolism in the brains of isoflurane-anesthetized and awake C57BL/6 N mice was compared using pulse-acquire sequences assessments. Both [1-^13^C]lactate to [1-^13^C]pyruvate and [^13^C]bicarbonate to [1-^13^C]pyruvate ratios were significantly higher under awake than isoflurane-anesthetized conditions (Figures 2C,D). One of the factors responsible may be increased CBF caused by isoflurane anesthesia. A previous study using slice-selective pulse imaging to compare the metabolic states of HP [1-^13^C]pyruvate in awake and anesthetized rat brains found that anesthesia-induced hemodynamic changes likely explain why the [1-^13^C]lactate to [1-^13^C]pyruvate and [^13^C]bicarbonate to [1-^13^C]pyruvate ratios were significantly higher in awake brains compared to those under isoflurane anesthesia (Hyppönen et al., 2022). The transport of HP [1-^13^C]pyruvate through the blood–brain barrier (BBB), with both active and passive components, has been identified as the rate-limiting step for ^13^C labeling of downstream metabolites (Hurd et al., 2010; Takado et al., 2018; Miller et al., 2018). In particular, because HP [1-^13^C]pyruvate transport through the BBB was expected to saturate throughout the experiment, the amount of HP [1-^13^C]pyruvate in the blood and the amount of blood in the voxel affected the observed apparent metabolite labeling rates (Josan et al., 2013). Thus, it is suggested that the apparent metabolic rate of pyruvate to its product is reduced in the brain under isoflurane anesthesia, as blood flow is increased (Hyppönen et al., 2022). The fact that CBF increased in the whole brain of C57BL/6 N mice under isoflurane-anesthetized conditions compared to awake conditions in the present study (Figure 4B) suggests the possibility that hemodynamics may influence the apparent labeling rate from HP [1-^13^C]pyruvate to metabolites observed in the pulse-acquire sequences assessment.

Furthermore, when the OFC and hippocampus of C57BL/6 N mice were evaluated separately, CBF was significantly increased in these regions under isoflurane-anesthetized conditions compared with the awake condition (Figure 4B). Thus, the fact that the [1-^13^C]lactate to [1-^13^C]pyruvate ratio was significantly higher in the VOIs that include the OFC or hippocampus of C57BL/6 N mice under awake conditions compared to isoflurane-anesthetized conditions in the present MRSI evaluation suggests that hemodynamic factors may influence the apparent rate of pyruvate-to-product metabolism (Figures 3C,D). This is consistent with findings from assessments using pulse-acquire sequences. In contrast, CBF alterations alone may not fully explain why the [^13^C]bicarbonate signal in the MRSI was undetectable in the VOIs that include the OFC and hippocampus of isoflurane-anesthetized C57BL/6 N mice but become detectable under awake conditions (Figure 3E). This suggests not only CBF alterations but also potential intracellular metabolic changes induced by anesthesia (Shulman et al., 1999). Supporting this, the [^13^C]bicarbonate to [1-^13^C]lactate ratio, which is considered less sensitive to HP [1-^13^C]pyruvate perfusion, was significantly altered between isoflurane-anesthetized and awake mouse brains in pulse-acquire sequences assessment (Figure 2E). Recent studies have reported that the brain uptake of ^18^F-FDG is lower in isoflurane-anesthetized mice than in awake mice and that high-concentration isoflurane anesthesia treatment induces a decrease in mitochondrial membrane potential levels involved in oxidative phosphorylation in mouse hippocampal neurons (Toyama et al., 2004; Zhang et al., 2012). Previous studies have indicated that glycolysis, lactate production, and respiration are stimulated in astrocytes in response to neuronal activation (Juaristi et al., 2019). Calcium signaling is the principal pathway through which astrocytes respond to neuronal activity, and isoflurane anesthesia has been shown to markedly suppress calcium transients in neocortical astrocytes (Thrane et al., 2012). Therefore, it may be important to include analysis under awake conditions, as in many clinical studies, in preclinical HP ^13^C MRS evaluations of changes in brain glycolysis and oxidative phosphorylation flux associated with physiological states and pathological conditions. On the other hand, HP [1-^13^C]pyruvate solution is typically administered to humans at doses of 35 mL or more in clinical studies (Larson et al., 2024), whereas in this study it was administered to mice at 10 μL/g body weight, approximately 10 times the dose by body weight. Given that the stress response to bolus infusion of HP solution under awake conditions may be greater in mice than in humans, further validation of the metabolic impact of the stress response in preclinical HP ^13^C MRS evaluation under awake conditions is warranted.

The present results comparing HP [1-^13^C]pyruvate metabolism in the brains of aged and young C57BL/6J mice using MRSI under awake conditions showed that the [^13^C] bicarbonate to [1-^13^C]pyruvate ratios in brain regions that include the hippocampus were significantly lower in aged mice than in young mice (Figure 5D). At that time, CBF in the whole brain, the OFC, and the hippocampus of awake C57BL/6J mice did not differ significantly between young and aged mice (Figure 5F), suggesting that the changes in the [^13^C]bicarbonate to [1-^13^C]pyruvate ratio shown in the MRSI may reflect differences in oxidative phosphorylation flux in brain regions that include the hippocampus between young and aged mice. A clinical study examining the changes in [1-^13^C]pyruvate uptake and metabolism in the human brain with aging showed that [^13^C]bicarbonate production decreases with age and that the rate of change varies by brain region (Uthayakumar et al., 2023). Our previous findings suggest that [1-^13^C]pyruvate metabolism, especially in oxidative phosphorylation flux, in the mouse brain reflects metabolic changes caused by calcium transients in astrocytes (Ono et al., 2024). Previous studies have shown that spatiotemporal reorganization of calcium ion events in mouse astrocytes and mitochondrial dysfunction in human astrocytes occurs with aging (Popov et al., 2021; Popov et al., 2023; Verkhratsky and Nedergaard, 2018). A recent study using a mouse model of pyruvate dehydrogenase deficiency in astrocytes demonstrated decreased bicarbonate signals following HP [1-^13^C]pyruvate injection, suggesting that astrocytes may be the source of bicarbonate derived from HP [1-^13^C]pyruvate (Marin-Valencia et al., 2024). Furthermore, clinical applications of proton-based MRS have recently revealed a correlation between elevated lactate concentrations and astrocyte activity markers in the brains of patients with neurodegenerative diseases (Hirata et al., 2024b). However, the results of CBF evaluation in the present study of awake C57BL/6J mice showed a trend toward greater individual differences in young mice than in aged mice (Figure 5F). Because the possibility of hemodynamic effects on the apparent labeling rate of HP [1-^13^C]pyruvate to metabolites remains to be considered, the relationship between HP [1-^13^C]pyruvate metabolism and CBF should be examined in the future by kinetic analysis using pulse-acquire sequences in awake C57BL/6J mice. In recent clinical studies, combined HP [1-^13^C]pyruvate and [^13^C, ^15^N_2_]urea MRS have been used for simultaneous metabolic and perfusion imaging (Qin et al., 2022; Liu et al., 2022), and it is important to investigate methods to simultaneously measure cerebral metabolism and CBF using mouse-awake MRSI. Currently, the low resolution of HP ^13^C MRSI presents a significant limitation. In this study, we used a method to detect metabolic changes by selecting voxels (2 × 2 × 8 mm^3^ per voxel) that predominantly contained specific brain regions. However, the selected VOIs also included signals from adjacent regions. Future advancements in hyperpolarization research could mitigate this issue by enabling smaller voxel sizes through approaches such as increasing sensitivity with cryo coils or optimizing ^13^C MRSI sequences. Despite this limitation, the awake MRSI for [1-^13^C]pyruvate metabolism established in this study holds significant potential for future applications in disease model mice. By integrating data on transporter expression levels and metabolic enzyme activity, these methods could substantially advance the analysis of abnormalities and alterations in glycolysis and oxidative phosphorylation flux, as well as their underlying mechanisms in neurodegenerative diseases and brain aging.

In conclusion, our results demonstrate the feasibility of spatial mapping of the metabolism of HP [1-^13^C]pyruvate to lactate and bicarbonate in awake mice. Furthermore, a decrease in oxidative phosphorylation flux in brain regions that include the hippocampus with aging was detected, and the extent of these changes varied across different brain regions. The present study demonstrated that HP [1-^13^C]pyruvate MRSI under awake conditions is useful for the spatial detection of abnormalities and alterations in glycolysis and oxidative phosphorylation flux in the brains of mice, which are currently widely used as disease model animals. Therefore, the use of HP [1-^13^C]pyruvate MRSI has potential in pathological and mechanistic studies of brain diseases and brain aging, as well as for the evaluation of disease-modifying drugs for neurodegenerative diseases.

## Data Availability

The raw data supporting the conclusions of this article will be made available by the authors, without undue reservation.

## References

[ref1] Ardenkjaer-Larsen J. H. Fridlund B. Gram A. Hansson G. Hansson L. Lerche M. H.. (2003). Increase in signal-to-noise ratio of > 10,000 times in liquid-state NMR. Proc. Natl. Acad. Sci. USA 100, 10158–10163. doi: 10.1073/pnas.1733835100, PMID: 12930897 PMC193532

[ref2] Chaumeil M. M. Bankson J. A. Brindle K. M. Epstein S. Gallagher F. A. Grashei M.. (2024). New horizons in hyperpolarized 13C MRI. Mol. Imaging Biol. 26, 222–232. doi: 10.1007/s11307-023-01888-5, PMID: 38147265 PMC10972948

[ref3] Choi Y.-S. Kang S. Ko S.-Y. Lee S. Kim J. Y. Lee H.. (2018). Hyperpolarized [1-13C] pyruvate MR spectroscopy detect altered glycolysis in the brain of a cognitively impaired mouse model fed high-fat diet. Mol. Brain 11:74. doi: 10.1186/s13041-018-0415-2, PMID: 30563553 PMC6299662

[ref4] Eichhorn T. R. Takado Y. Salameh N. Capozzi A. Cheng T. Hyacinthe J. N.. (2013). Hyperpolarization without persistent radicals for *in vivo* real-time metabolic imaging. Proc. Natl. Acad. Sci. USA 110, 18064–18069. doi: 10.1073/pnas.1314928110, PMID: 24145405 PMC3831441

[ref5] Grist J. T. McLean M. A. Riemer F. Schulte R. F. Deen S. S. Zaccagna F.. (2019). Quantifying normal human brain metabolism using hyperpolarized [1–^13^C]pyruvate and magnetic resonance imaging. NeuroImage 189, 171–179. doi: 10.1016/j.neuroimage.2019.01.027, PMID: 30639333 PMC6435102

[ref6] Grist J. T. Miller J. J. Zaccagna F. McLean M. A. Riemer F. Matys T.. (2020). Hyperpolarized ^13^C MRI: a novel approach for probing cerebral metabolism in health and neurological disease. J. Cereb. Blood Flow Metab. 40, 1137–1147. doi: 10.1177/0271678X20909045, PMID: 32153235 PMC7238376

[ref7] Hackett E. P. Pinho M. C. Harrison C. E. Reed G. D. Liticker J. Raza J.. (2020). Imaging acute metabolic changes in patients with mild traumatic brain injury using hyperpolarized [1–^13^C] pyruvate. iScience 23:101885. doi: 10.1016/j.isci.2020.101885, PMID: 33344923 PMC7736977

[ref8] Hirata K. Matsuoka K. Tagai K. Endo H. Tatebe H. Ono M.. (2024a). *In vivo* assessment of astrocyte reactivity in patients with progressive supranuclear palsy. Ann. Neurol. 96, 247–261. doi: 10.1002/ana.26962, PMID: 38771066

[ref9] Hirata K. Matsuoka K. Tagai K. Endo H. Tatebe H. Ono M.. (2024b). Altered brain energy metabolism related to astrocytes in Alzheimer's disease. Ann. Neurol. 95, 104–115. doi: 10.1002/ana.26797, PMID: 37703428

[ref10] Hurd R. E. Yen Y.-F. Tropp J. Pfefferbaum A. Spielman D. M. Mayer D. (2010). Cerebral dynamics and metabolism of hyperpolarized [1-(13)C]pyruvate using time-resolved MR spectroscopic imaging. J. Cereb. Blood Flow Metab. 30, 1734–1741. doi: 10.1038/jcbfm.2010.93, PMID: 20588318 PMC2975615

[ref11] Hyppönen V. Stenroos P. Nivajärvi R. Ardenkjaer-Larsen J. H. Gröhn O. Paasonen J.. (2022). Metabolism of hyperpolarised [1-^13^ C]pyruvate in awake and anaesthetised rat brains. NMR Biomed. 35:e4635. doi: 10.1002/nbm.4635, PMID: 34672399

[ref12] Josan S. Hurd R. Billingsley K. Senadheera L. Park J. M. Yen Y.-F.. (2013). Effects of isoflurane anesthesia on hyperpolarized (13)C metabolic measurements in rat brain. Magn. Reson. Med. 70, 1117–1124. doi: 10.1002/mrm.24532, PMID: 23086864 PMC3674171

[ref13] Juaristi I. Contreras L. González-Sánchez P. Pérez-Liébana I. González-Moreno L. Pardo B.. (2019). The response to stimulation in neurons and astrocytes. Neurochem. Res. 44, 2385–2391. doi: 10.1007/s11064-019-02803-7, PMID: 31016552

[ref14] Larson E. Z. P. Bernard M. L. J. Bankson A. J. Bøgh N. Bok A. R. Chen P. A.. (2024). Current methods for hyperpolarized [1-^13^C]pyruvate MRI human studies. Magn. Reson. Med. 91, 2204–2228. doi: 10.1002/mrm.29875, PMID: 38441968 PMC10997462

[ref15] Le Page L. M. Guglielmetti C. Taglang C. Chaumeil M. M. (2020). Imaging brain metabolism using hyperpolarized ^13^C magnetic resonance spectroscopy. Trends Neurosci. 43, 343–354. doi: 10.1016/j.tins.2020.03.006, PMID: 32353337 PMC7255622

[ref16] Lee C. Y. Soliman H. Geraghty B. J. Chen A. P. Connelly K. A. Endre R.. (2020). Lactate topography of the human brain using hyperpolarized ^13^C-MRI. NeuroImage 204:116202. doi: 10.1016/j.neuroimage.2019.116202, PMID: 31557546

[ref17] Li Y. Vigneron D. B. Xu D. (2021). Current human brain applications and challenges of dynamic hyperpolarized carbon-13 labeled pyruvate MR metabolic imaging. Eur. J. Nucl. Med. Mol. Imaging 48, 4225–4235. doi: 10.1007/s00259-021-05508-8, PMID: 34432118 PMC8566394

[ref18] Liu X. Tang S. Mu C. Qin H. Cui D. Lai Y.-C.. (2022). Development of specialized magnetic resonance acquisition techniques for human hyperpolarized [13 C,15 N2]urea + [1-13 C]pyruvate simultaneous perfusion and metabolic imaging. Magn. Reson. Med. 88, 1039–1054. doi: 10.1002/mrm.29266, PMID: 35526263 PMC9810116

[ref19] Lukasik V. M. Gillies R. J. (2003). Animal anaesthesia for *in vivo* magnetic resonance. NMR Biomed. 16, 459–467. doi: 10.1002/nbm.836, PMID: 14696002

[ref20] Marin-Valencia I. Kocabas A. Rodriguez-Navas C. Miloushev V. Z. González-Rodríguez M. Lees H.. (2024). Imaging brain glucose metabolism *in vivo* reveals propionate as a major anaplerotic substrate in pyruvate dehydrogenase deficiency. Cell Metab. 36, 1394–1410.e12. doi: 10.1016/j.cmet.2024.05.002, PMID: 38838644 PMC11187753

[ref21] Miller J. J. Grist J. T. Serres S. Larkin J. R. Lau A. Z. Ray K.. (2018). ^13^C pyruvate transport across the blood-brain barrier in preclinical hyperpolarised MRI. Sci. Rep. 8:15082. doi: 10.1038/s41598-018-33363-5, PMID: 30305655 PMC6180068

[ref22] Ono M. Wulaer B. Yamasaki T. Sakamoto T. Araki R. Hirata K.. (2024). Astrocytes contribute to signals of hyperpolarized 13C pyruvate in the brain. ISMRM & ISMRT Annual meeting & exhibition, 10.

[ref23] Park I. Larson P. E. Z. Gordon J. W. Carvajal L. Chen H. Y. Bok R.. (2018). Development of methods and feasibility of using hyperpolarized carbon-13 imaging data for evaluating brain metabolism in patient studies. Magn. Reson. Med. 80, 864–873. doi: 10.1002/mrm.27077, PMID: 29322616 PMC5980662

[ref24] Popov A. Brazhe A. Denisov P. Sutyagina O. Li L. Lazareva N.. (2021). Astrocyte dystrophy in ageing brain parallels impaired synaptic plasticity. Aging Cell 20:e13334. doi: 10.1111/acel.13334, PMID: 33675569 PMC7963330

[ref25] Popov A. Brazhe N. Morozova K. Yashin K. Bychkov M. Nosova O.. (2023). Mitochondrial malfunction and atrophy of astrocytes in the aged human cerebral cortex. Nat. Commun. 14:8380. doi: 10.1038/s41467-023-44192-0, PMID: 38104196 PMC10725430

[ref26] Qin H. Tang S. Riselli A. M. Bok R. A. Santos R. D. van Criekinge M.. (2022). Clinical translation of hyperpolarized 13 C pyruvate and urea MRI for simultaneous metabolic and perfusion imaging. Magn. Reson. Med. 87, 138–149. doi: 10.1002/mrm.28965, PMID: 34374471 PMC8616838

[ref27] Shulman R. G. Rothman D. L. (2001). 13C NMR of intermediary metabolism: implications for systemic physiology. Annu. Rev. Physiol. 63, 15–48. doi: 10.1146/annurev.physiol.63.1.15, PMID: 11181947

[ref28] Shulman R. G. Rothman D. L. Hyder F. (1999). Stimulated changes in localized cerebral energy consumption under anesthesia. Proc. Natl. Acad. Sci. USA 96, 3245–3250. doi: 10.1073/pnas.96.6.3245, PMID: 10077669 PMC15927

[ref29] Sumiyoshi A. Shibata S. Zhelev Z. Miller T. Lazarova D. Aoki I.. (2022). Targeting glioblastoma via selective alteration of mitochondrial redox state. Cancers (Basel) 14:485. doi: 10.3390/cancers14030485, PMID: 35158753 PMC8833725

[ref30] Takado Y. Cheng T. Bastiaansen J. A. M. Yoshihara H. A. I. Lanz B. Mishkovsky M.. (2018). Hyperpolarized ^13^C magnetic resonance spectroscopy reveals the rate-limiting role of the blood_brain barrier in the cerebral uptake and metabolism of L-lactate *in vivo*. ACS Chem. Neurosci. 9, 2554–2562. doi: 10.1021/acschemneuro.8b00066, PMID: 29771492 PMC6119468

[ref31] Thrane A. S. Thrane V. R. Zeppenfeld D. Lou N. Xu Q. Nagelhus E. A.. (2012). General anesthesia selectively disrupts astrocyte calcium signaling in the awake mouse cortex. Proc. Natl. Acad. Sci. USA 109, 18974–18979. doi: 10.1073/pnas.1209448109, PMID: 23112168 PMC3503159

[ref32] Toyama H. Ichise M. Liow J. S. Vines D. C. Seneca N. M. Modell K. J.. (2004). Evaluation of anesthesia effects on [18F]FDG uptake in mouse brain and heart using small animal PET. Nucl. Med. Biol. 31, 251–256. doi: 10.1016/S0969-8051(03)00124-0, PMID: 15013491

[ref33] Uthayakumar B. Soliman H. Bragagnolo N. D. Cappelletto N. I. C. Lee C. Y. Geraghty B.. (2023). Age-associated change in pyruvate metabolism investigated with hyperpolarized ^13^ C-MRI of the human brain. Hum. Brain Mapp. 44, 4052–4063. doi: 10.1002/hbm.26329, PMID: 37219519 PMC10258534

[ref34] Verkhratsky A. Nedergaard M. (2018). Physiology of Astroglia. Physiol. Rev. 98, 239–389. doi: 10.1152/physrev.00042.2016, PMID: 29351512 PMC6050349

[ref35] Zhang Y. Xu Z. Wang H. Dong Y. Shi H. N. Culley D. J.. (2012). Anesthetics isoflurane and desflurane differently affect mitochondrial function, learning, and memory. Ann. Neurol. 71, 687–698. doi: 10.1002/ana.23536. Epub 2012 Feb 24, PMID: 22368036 PMC3942786

