# Supplementary material for: Awake brain MRSI reveals anesthetic sensitivity and regional aging effects on [13C]bicarbonate metabolism in mice
Source: Front Neuroimaging. 2025 Feb 12;4:1506126. doi: 10.3389/fnimg.2025.1506126 (PMC11861090; doi:10.3389/fnimg.2025.1506126)
Supplement: Supplementary file 1 [file Data_Sheet_1.pdf]

Supplementary Material

1.1 Supplementary Figures

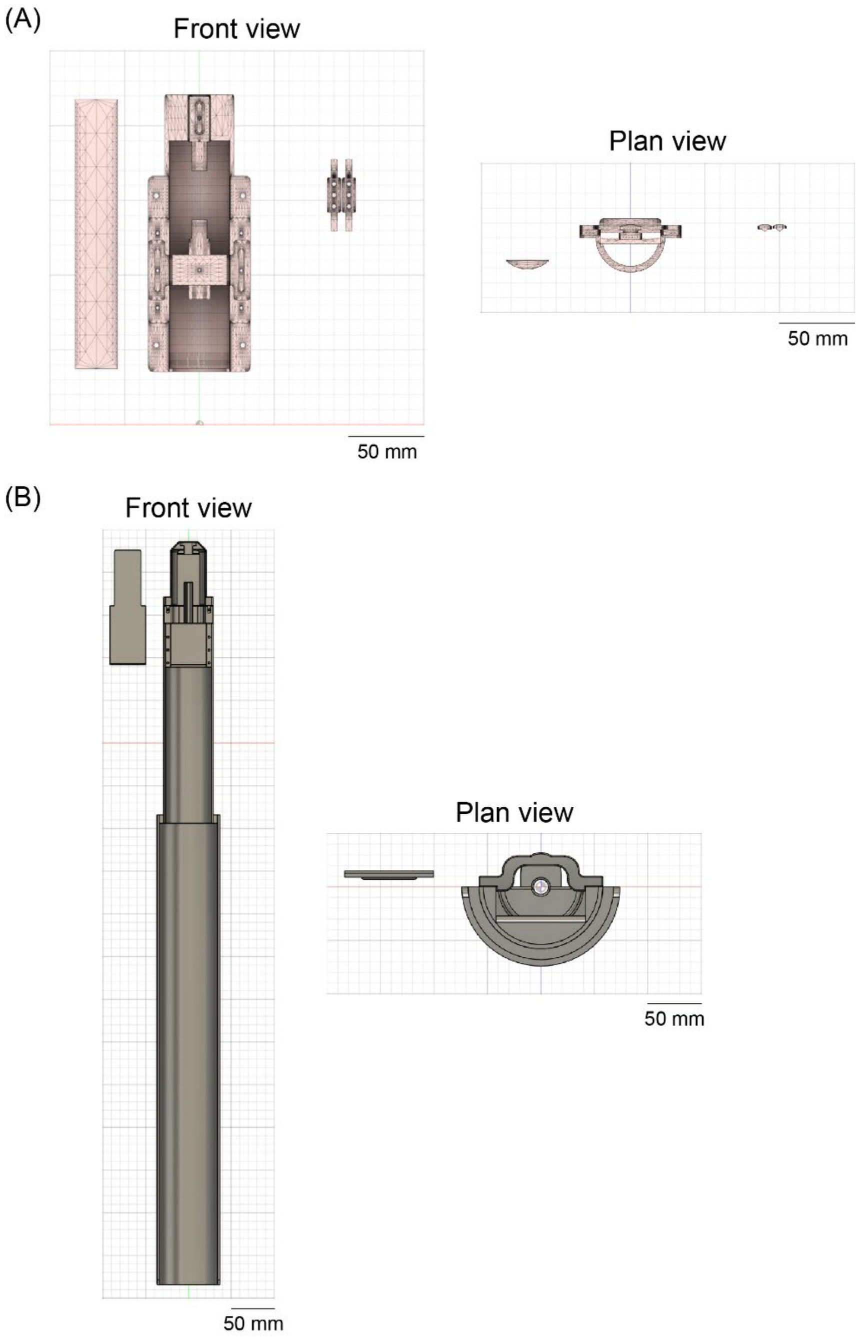

Supplementary Figure 1. (A and B) Designs of a custom-made awake-mouse restraint device for a 3T (A) and 7T (B) preclinical MR scanner.

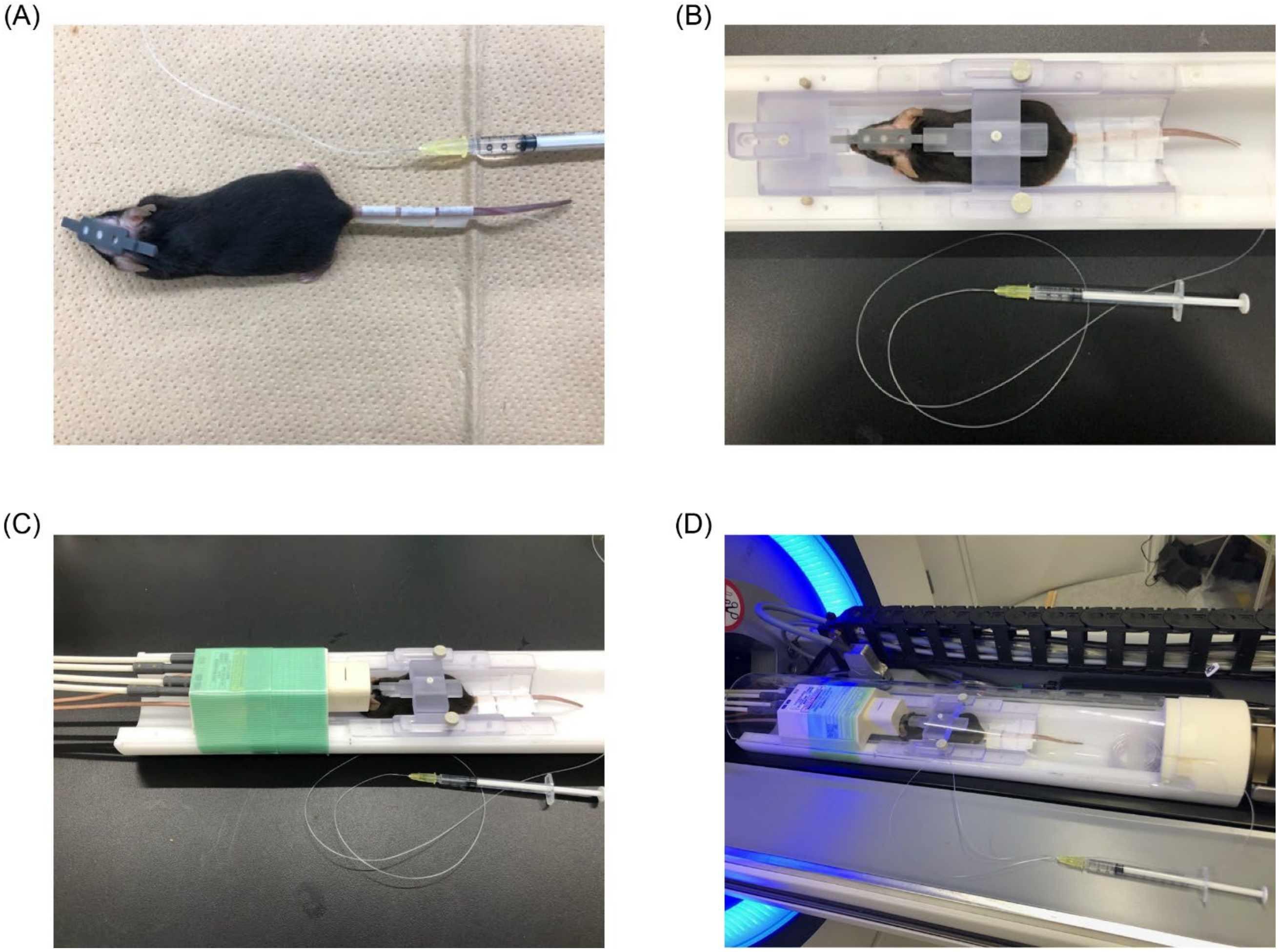

Supplementary Figure 2. (A-C) Instructions for using the custom-made awake-mouse restraint device. The animals were anesthetized with 1.5% (w/v) isoflurane, and a tail vein catheter was inserted for the injection of hyperpolarized [1-^{13}C]pyruvate (A). The mice were then secured in the custom-made awake-mouse restraint device via the headplate (B). After positioning, the ^{1}H-^{13}C dual-tuned surface transmit/receive coil was placed above the mouse's head (C), and the entire setup, including the restraint device, was inserted into the scanner (D).

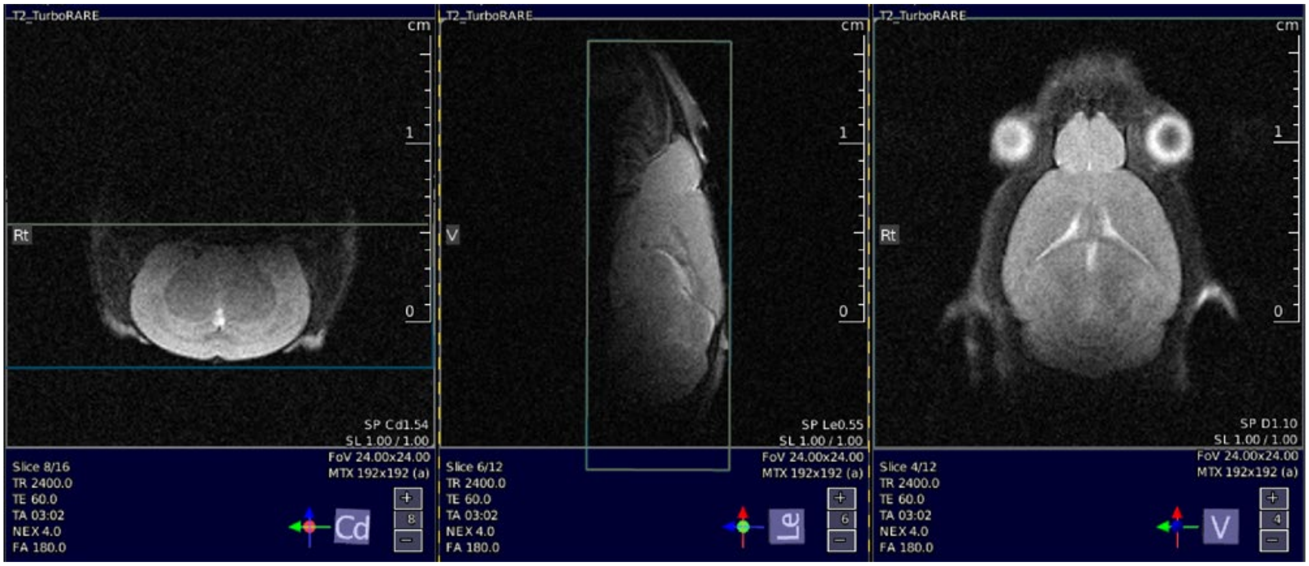

Supplementary Figure 3. Slab for MRSI.

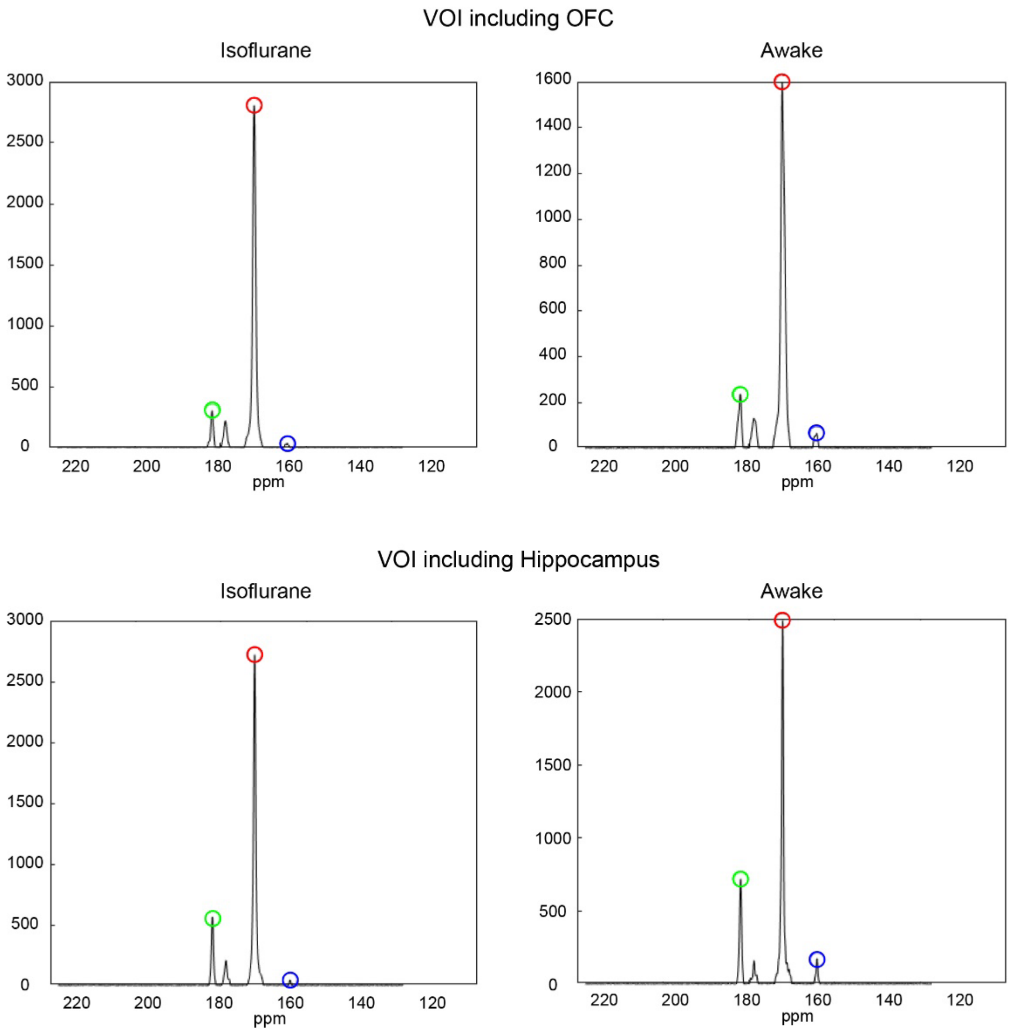

Supplementary Figure 4. *In vivo* real-time pyruvate metabolism evaluated using MRSI in isoflurane-anesthetized and awake C57BL/6N mouse brains. *In vivo* ^{13}C MR data recorded following the injection of a [1-^{13}C]pyruvate solution (80.7 mmol/L, 10 µL/g body weight). The data were analyzed in absorption mode using custom-built programs written in MATLAB (MathWorks, Natick, MA, USA). Specifically, FID data were Fourier transformed, and 0th and 1st-order phase correction factors were manually determined using a voxel with a sufficiently strong signal. These phase correction factors were then applied to all voxels. A spline function was fitted to the region outside the peaks and subtracted from the absorption spectrum. The integral of each peak was calculated for quantification. The sum of all five ^{13}C spectra acquired after administration of [1-^{13}C]pyruvate in the VOIs that include the OFC (upper) and hippocampus (lower) of 2-month-old isoflurane-anesthetized and awake C57BL/6N mice is displayed. Peaks corresponding to lactate, pyruvate, and bicarbonate are marked with green, red, and blue circles, respectively.

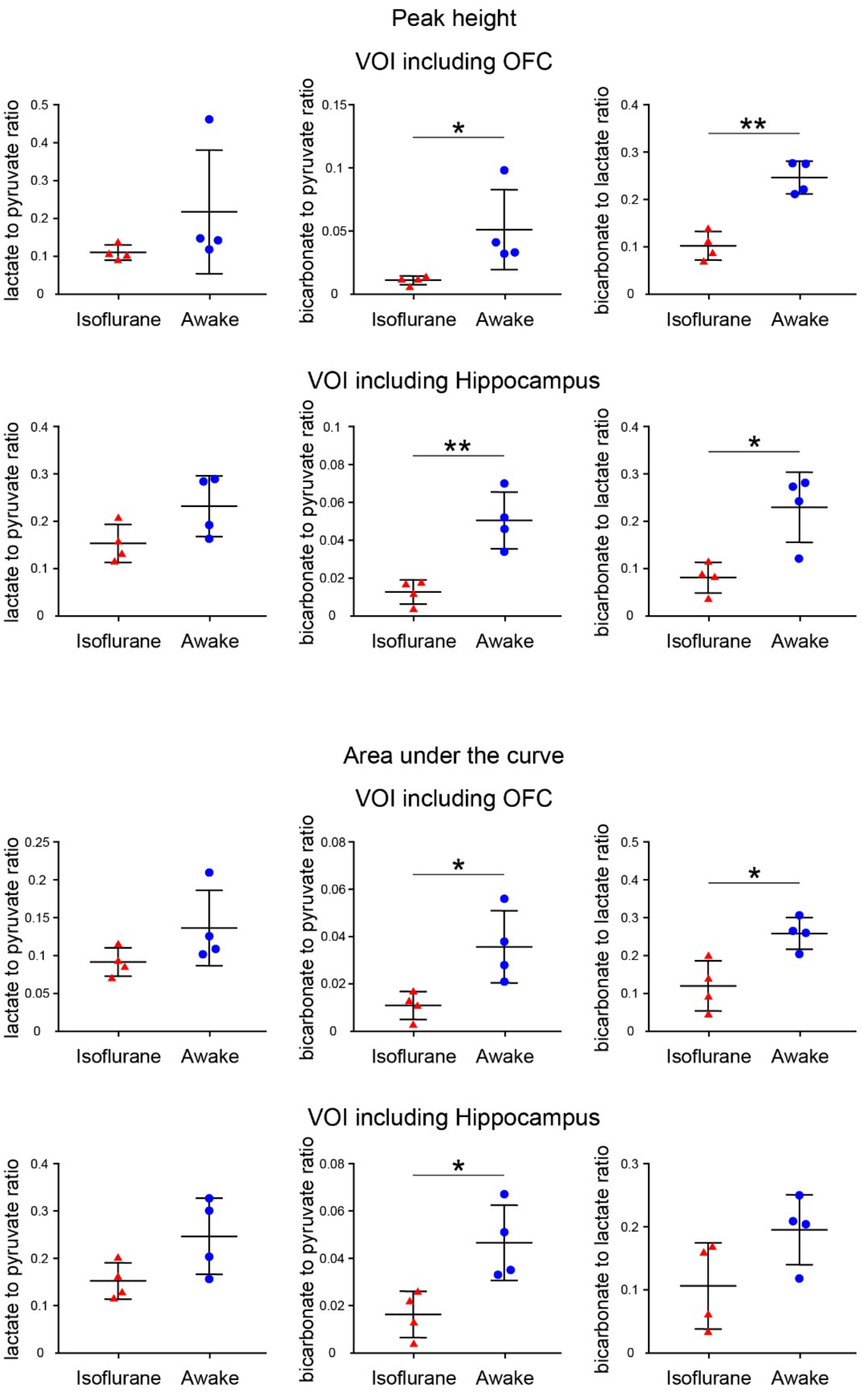

Supplementary Figure 5. The [1-^{13}C]lactate to [1-^{13}C]pyruvate ratio (left), the [^{13}C]bicarbonate to [1-^{13}C]pyruvate ratio (middle), and the [^{13}C]bicarbonate to [1-^{13}C]lactate ratio (right) in the VOIs that include the OFC and hippocampus of 2-month-old isoflurane-anesthetized (n = 4) and awake (n = 4) C57BL/6N mice were quantified on respective peak heights and area under the curves from the MRSI data analyzed in absorption mode. *$p < 0.05$, **$p < 0.005$

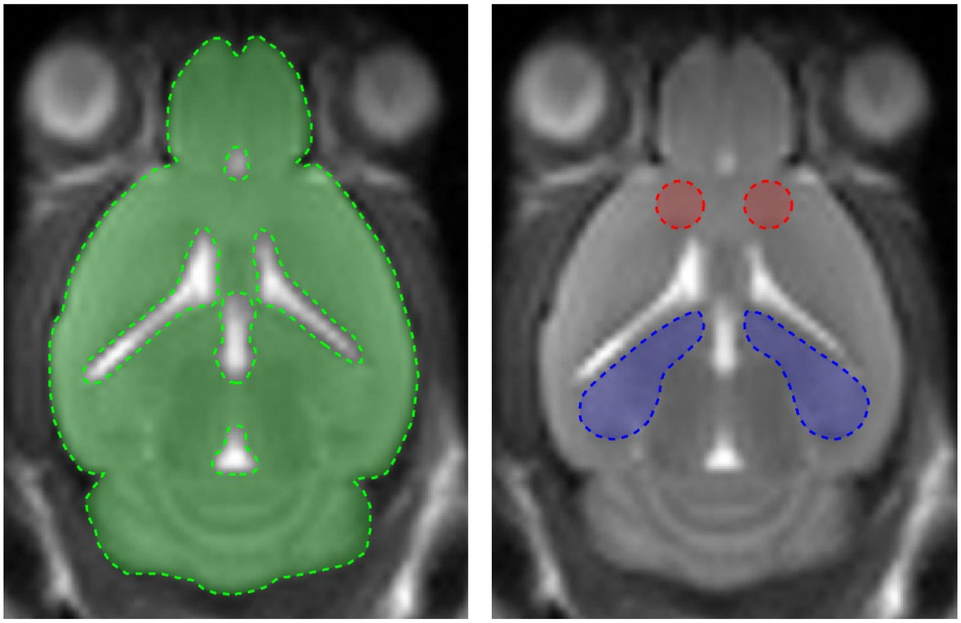

Supplementary Figure 6. Horizontal FAIR image of C57BL/6N mouse brain. Areas painted green, red, and blue depict representative ROIs of the whole brain, OFC, and hippocampus, respectively.

1.2 Supplementary Tables

Detailed information on mice used in the experiments.

Pulse-acquire sequences assessment (Figure 2)

Isoflurane-anesthetized condition

Mouse No.	Strain	Sex	Age in months at time of experiment
# 1	C57BL/6N	Male	2 month old
# 2	C57BL/6N	Male	2 month old
# 3	C57BL/6N	Male	2 month old
# 4	C57BL/6N	Male	2 month old
# 5	C57BL/6N	Male	2 month old

* Data from mouse #5 was excluded from the analysis due to low S/N.

Awake condition

Mouse No.	Strain	Sex	Age in months at time of experiment
# 1	C57BL/6N	Male	2 month old
# 2	C57BL/6N	Male	2 month old
# 3	C57BL/6N	Male	2 month old
# 4	C57BL/6N	Male	2 month old
# 5	C57BL/6N	Male	2 month old

CSI evaluation (Figure 3)

Isoflurane-anesthetized condition

Mouse No.	Strain	Sex	Age in months at time of experiment
# 5	C57BL/6N	Male	2 month old
# 6	C57BL/6N	Male	2 month old
# 7	C57BL/6N	Male	2 month old
# 8	C57BL/6N	Male	2 month old

Awake condition

Mouse No.	Strain	Sex	Age in months at time of experiment
# 5	C57BL/6N	Male	2 month old
# 6	C57BL/6N	Male	2 month old
# 7	C57BL/6N	Male	2 month old
# 8	C57BL/6N	Male	2 month old

Cerebral perfusion measurements (Figure 4)

Isoflurane-anesthetized condition

Mouse No.	Strain	Sex	Age in months at time of experiment
# 5	C57BL/6N	Male	4 month old
# 7	C57BL/6N	Male	4 month old
# 8	C57BL/6N	Male	4 month old
# 9	C57BL/6N	Male	4 month old
# 10	C57BL/6N	Male	4 month old

Awake condition

Mouse No.	Strain	Sex	Age in months at time of experiment
# 5	C57BL/6N	Male	4 month old
# 7	C57BL/6N	Male	4 month old
# 8	C57BL/6N	Male	4 month old
# 9	C57BL/6N	Male	4 month old
# 10	C57BL/6N	Male	4 month old

CSI evaluation (Figure 5)

Aged mice

Mouse No.	Strain	Sex	Age in months at time of experiment
# 11	C57BL/6J	Male	19 month old
# 12	C57BL/6J	Male	19 month old
# 13	C57BL/6J	Male	19 month old
# 14	C57BL/6J	Male	19 month old
# 15	C57BL/6J	Male	19 month old
# 16	C57BL/6J	Male	19 month old
# 17	C57BL/6J	Male	19 month old
# 18	C57BL/6J	Male	19 month old
# 19	C57BL/6J	Male	19 month old

Young mice

Mouse No.	Strain	Sex	Age in months at time of experiment
# 20	C57BL/6J	Male	2.5 month old
# 21	C57BL/6J	Male	2.5 month old
# 22	C57BL/6J	Male	2.5 month old
# 23	C57BL/6J	Male	2.5 month old
# 24	C57BL/6J	Male	2.5 month old
# 25	C57BL/6J	Male	2.5 month old
# 26	C57BL/6J	Male	2.5 month old
# 27	C57BL/6J	Male	2.5 month old
# 28	C57BL/6J	Male	2.5 month old

Cerebral perfusion measurements (Figure 5)

Aged mice

Mouse No.	Strain	Sex	Age in months at time of experiment
# 11	C57BL/6J	Male	19 month old
# 12	C57BL/6J	Male	19 month old
# 13	C57BL/6J	Male	19 month old
# 14	C57BL/6J	Male	19 month old
# 15	C57BL/6J	Male	19 month old
# 16	C57BL/6J	Male	19 month old
# 17	C57BL/6J	Male	19 month old
# 18	C57BL/6J	Male	19 month old
# 19	C57BL/6J	Male	19 month old

Young mice

Mouse No.	Strain	Sex	Age in months at time of experiment
# 20	C57BL/6J	Male	2.5 month old
# 21	C57BL/6J	Male	2.5 month old
# 22	C57BL/6J	Male	2.5 month old
# 23	C57BL/6J	Male	2.5 month old
# 24	C57BL/6J	Male	2.5 month old
# 25	C57BL/6J	Male	2.5 month old
# 26	C57BL/6J	Male	2.5 month old
# 27	C57BL/6J	Male	2.5 month old
# 28	C57BL/6J	Male	2.5 month old
